# Role of lymphatic invasion in predicting biochemical recurrence after radical prostatectomy

**DOI:** 10.3389/fonc.2023.1226366

**Published:** 2023-09-11

**Authors:** Dae Hyuk Chung, Jang Hee Han, Seung-Hwan Jeong, Hyeong Dong Yuk, Chang Wook Jeong, Ja Hyeon Ku, Cheol Kwak

**Affiliations:** ^1^ Department of Urology, Seoul National University Hospital, Seoul, Republic of Korea; ^2^ Department of Urology, Seoul National University College of Medicine, Seoul, Republic of Korea

**Keywords:** prostate cancer, lymphatic invasion, lymph node metastasis, biochemical recurrence, radical prostatectomy

## Abstract

**Objective:**

Lymphatic invasion in prostate cancer is associated with poor prognosis. However, there is no consensus regarding the clinical and prognostic value of lymphatic invasion. This study aimed to investigate the prognostic value of lymphatic invasion in biochemical recurrence (BCR) and compare the recurrence rates between patients with lymphatic invasion and lymph node metastasis.

**Methods:**

We retrospectively analyzed 2,207 patients who underwent radical prostatectomy (RP) without pelvic lymph node dissection (PLND) and 742 patients who underwent RP with PLND for clinically localized or locally advanced prostate cancer, between 1993 and 2020, at Seoul National University Hospital. Kaplan–Meier analysis was performed to estimate BCR-free survival (BCRFS) using the log-rank test. The Cox proportional hazards model was used to identify the significant factors for BCR. Propensity score matching was performed with a 1:2 ratio to match age, initial PSA level, pathological T stage, and Gleason score to exclude confounding effects.

**Results:**

Of the 2,207 patients who underwent RP without PLND, lymphatic invasion (L1Nx) was observed in 79 (3.5%) individuals. Among the 742 patients who underwent RP with PLND, lymph node metastases were found in 105 patients (14.2%). In patients with lymph node metastasis, lymphatic invasion was observed in 50 patients (47.6%), whereas lymphatic invasion was observed in 53 patients (8.3%) among those without lymph node metastasis. In patients who underwent RP without PLND, Kaplan–Meier analysis showed significantly poorer BCR-free survival in the L1Nx group than in the L0Nx group (p < 0.001). In patients who underwent RP with PLND, the L1N0, L0N1, and L1N1 groups showed significantly worse prognoses than the L0N0 group (p < 0.001). However, there was no significant difference in BCRFS between the L1N0 and lymph node metastasis groups, including the L0N1 and L1N1 groups. After propensity score matching at a 1:2 ratio, the L1Nx group showed significantly poorer outcomes in terms of BCRFS than the L0Nx group (p = 0.05). In addition, the L1N0 group showed a significantly worse prognosis than the L0N0 group after propensity score matching.

**Conclusion:**

Lymphatic invasion in radical prostatectomy specimens is an independent prognostic factor, which can complement lymph node status for predicting biochemical recurrence. Considering lymphatic invasion as an adverse pathological finding, similar to lymph node metastasis, adjuvant therapy could be considered in patients with lymphatic invasion.

## 1 Introduction

Lymph nodes near the primary cancer are considered the first site of metastasis (1, 2), and the existence of lymph node metastasis is known to be a crucial prognostic factor in many cancers (3–5). Patients with prostate cancer with lymph node metastasis have a poor prognosis (6, 7). Several retrospective studies have demonstrated that pathological N1 stage (N1) after radical prostatectomy (RP) with pelvic lymph node dissection (PLND) is a risk factor for recurrence (8, 9). To reduce relapse in patients with N1 disease after RP with PLND, current guidelines suggest androgen deprivation therapy with or without radiation therapy after surgery (10, 11).

Similar to that in other cancers, lymphatic invasion in prostate cancer is associated with lymph node metastasis (12). Several retrospective studies have revealed that lymphatic invasion (L1) in radical prostatectomy specimens is a risk factor for biochemical recurrence (BCR) (13, 14). Therefore, the International Collaboration on Cancer Reporting (ICCR) recommends providing information regarding the presence of lymphovascular invasion in radical prostatectomy specimens (15).

Although L1 is associated with poor prognosis in prostate cancer, there is no definite consensus on the clinical and prognostic value of lymphatic invasion. The aim of our study was to evaluate the prognostic value of L1 and determine whether L1 can be a complement or substitute for lymph node status in predicting recurrence.

## 2 Materials and methods

### 2.1 Ethics approval and informed consent

This study was approved by the Institutional Review Board (IRB No. H-305-041-1430) of Seoul National University Hospital. The requirement for informed consent was waived due to the retrospective nature of the study. All the research and related protocols used in this study were performed in accordance with the principles of the Declaration of Helsinki.

### 2.2 Patient selection

We conducted a retrospective study of 2,207 patients who underwent RP without PLND and 742 patients who underwent RP with PLND for clinically localized or locally advanced prostate cancer between 1993 and 2020 at the Seoul National University Hospital. The clinical data of these patients were acquired retrospectively by reviewing their electronic medical records. All surgeries were performed by several surgeons at a single center using one of the following techniques: open, laparoscopic, or robot-assisted laparoscopy. PLND was performed according to risk stratification or clinical stage, and all PLND was performed in standard extent.

All tumor-containing blocks were analyzed by experienced genitourinary pathologists according to standard procedures, including complete embedding of the entire prostate. The histopathological findings from the resected specimens included Gleason score, pathological T stage, pathological N stage, lymphatic invasion, venous invasion, and perineural invasion. Unlike other institutes that only report lymphovascular invasion from specimens, we separately analyzed lymphatic and venous invasions.

We retrospectively collected clinical data from patients, including age, Eastern Cooperative Oncology Group (ECOG) performance status grade, and initial serum prostate-specific antigen (PSA) level, which was defined as the PSA value just before prostate biopsy. Postoperative serum PSA levels were measured every 3 months, 2 years after surgery, and every 6 months thereafter. BCR was defined as a detectable or rising PSA ≥0.2 ng/ml, followed by a confirmatory PSA of ≥0.2 ng/ml. All patients with PSA persistence after radical prostatectomy were excluded from the study. Adjuvant radiation therapy or adjuvant androgen deprivation therapy (ADT) was administered to patients with adverse pathological factors, including positive surgical margins and N1 stage.

### 2.3 Statistical analysis

All continuous variables were described as mean ± standard deviation, whereas categorical variables were described as frequency (percentage). Categorical variables were analyzed using chi-square tests and numerical variables were compared using a two-tailed Student’s t-test. Kaplan–Meier analysis was performed to estimate BCR-free survival using the log-rank test for significance assessment.

To verify the independence of lymphatic invasion as a factor for BCR, propensity score matching was performed with a 1:2 ratio to match age, initial PSA level, pathological T stage, and Gleason score to exclude confounding effects. The Cox proportional hazards model was used to identify significant factors for BCR. All statistical analyses were performed using XLSTAT (version 2021.5-Life Sciences). Statistical significance was set at p < 0.05.

## 3 Results

### 3.1 Clinicopathological characteristics of the patients

In total, 2,207 patients underwent RP without PLND, and lymphatic invasion (L1Nx) was observed in 79 patients (3.5%). **Table 1** compares the clinicopathological features between with lymphatic invasion (L1Nx) and without lymphatic invasion (L0Nx) in patients who underwent RP without PLND. The initial serum PSA level was significantly higher in the L1Nx group than in the L0Nx group (13.89 versus 9.28, p < 0.0001). Furthermore, the pathological T stage and Gleason score tended to be higher in L1Nx patients, indicating adverse pathological features (p < 0.0001). Venous invasion, perineural invasion, and positive surgical margin were also higher in the L1Nx group than in the L0Nx group (p < 0.0001). Adjuvant radiation therapy was administered to eight patients (0.4%) in the L0Nx group, but none in the L1Nx group. Adjuvant ADT was given to 41 patients (1.9%) in the L0Nx group but four patients (5.1%) in the L1Nx group. However, there was no significant difference in the proportion of adjuvant therapy between L0Nx and L1Nx group.

**Table 1:** Clinicopathological data between with lymphatic invasion (L1Nx) and without lymphatic invasion (L0Nx) in patients who underwent radical prostatectomy (RP) without pelvic lymph node dissection (PLND).

	L0Nx (n = 2,128)	L1Nx (n = 79)	p-value
Age (mean)	66.7 ± 6.5	66.9 ± 8.2	0.768
iPSA	9.28 ± 8.84	13.89 ± 13.08	**<0.0001**
ECOG score 0 1 2	2,111 (99.2) 7 (0.3) 10 (0.5)	79 (100) 0 0	0.537
Operation type Open Laparoscopic Robotic	848 (39.8) 50 (2.3) 1,230 (57.8)	36 (45.6) 0 43 (54.4)	0.111
pT stage 2a 2b 2c 3a 3b 4	361 (0.1) 57 (30.5) 1,024 (62.9) 550 (3.6) 132 (2.8) 4 (0.1)	4 (5.1) 1 (1.3) 17 (21.5) 32 (40.5) 25 (31.6) 0	**<0.0001**
Gleason Score 6 7 8 9 10	649 (30.5) 1,341 (62.9) 77 (3.6) 59 (2.8) 2 (0.1)	0 54 (68.4) 5 (6.3) 18 (22.8) 2 (2.59)	**<0.0001**
Positive surgical margin	632 (29.7)	43 (54.4)	**<0.0001**
Venous invasion	10 (0.5)	5 (6.3)	**<0.0001**
Perineural invasion	1,027 (48.3)	61 (78.5)	**<0.0001**
Adjuvant radiation therapy	8 (0.4)	0	0.445
Adjuvant ADT	41 (1.9)	4 (5.1)	0.1

P-values in bold font indicate statistical significance (P-value <0.05).

Among the 742 patients who underwent RP with PLND, lymph node metastases were found in 105 patients (14.2%). In patients with lymph node metastasis, lymphatic invasion was observed in 50 patients (47.6%), whereas lymphatic invasion was observed in 53 patients (8.3%) among those without lymph node metastasis. **Table 2** shows a comparison of clinicopathological features in pN0 patients between with lymphatic invasion (L1N0) and without lymphatic invasion (L0N0). The mean age was significantly higher in the L1N0 group than that in the L0N0 group (66.3 versus 68.3 years, p = 0.038). As shown in **Table 2**, the initial serum PSA level, pathological T stage, Gleason score, venous invasion, perineural invasion, and positive surgical margin in the L1N0 group were also significantly higher in the L1N0 group compared with L0N0 group (all p < 0.001). Adjuvant radiation therapy was given to nine patients (1.5%) in the L0N0 group but none in the L1N0 group. Adjuvant ADT was administered to 16 patients (2.7%) in the L0N0 group but three patients (5.7%) in the L1N0 group. However, there was also no significant difference in the proportion of adjuvant therapy between L0N0 and L1N0 group. **Table 3** shows a comparison of clinicopathological features among the L1N0, L0N1, and L1N1 groups. There were significant differences only in the initial serum PSA level (p = 0.002) and the number of patients who received adjuvant ADT (p = 0.012).

**Table 2:** Clinicopathological data between with lymphatic invasion (L1N0) and without lymphatic invasion (L0N0) in pathological node-negative patients who underwent RP with PLND.

	L0N0 (n = 584)	L1N0 (n = 53)	p-value
Age (mean)	66.3 ± 7.2	68.3 ± 5.8	0.038
iPSA	15.4 ± 21.2	19.2 ± 13.2	**<0.001**
ECOG score 0 1 2	580 (99.3) 1 (0.2) 3 (0.5)	51 (96.2) 1 (1.9) 1 (1.9)	0.181
Operation type Open Laparoscopic Robotic	316 (54.1) 5 (0.9) 263 (45.0)	20 (37.7) 2 (3.8) 31 (58.5)	0.031
pT stage 2a 2b 2c 3a 3b 4	60 (10.3) 7 (1.2) 222 (38.0) 198 (33.9) 95 (16.3) 2 (0.3)	1 (1.9) 0 2 (3.8) 20 (37.7) 29 (54.7) 1 (1.9)	**<0.0001**
Gleason Score 6 7 8 9 10	68 (11.6) 396 (67.8) 59 (10.1) 60 (10.3) 1 (0.2)	3 (5.7) 23 (43.4) 9 (17.0) 17 (32.1) 1 (1.9)	**<0.0001**
Positive surgical margin	237 (40.6)	37 (69.8)	**<0.0001**
Venous invasion	4 (0.7)	6 (11.3)	**<0.0001**
Perineural invasion	347 (58.4)	47 (88.7)	**<0.0001**
Adjuvant radiation therapy	9 (1.5)	0	0.209
Adjuvant ADT	16 (2.7)	3 (5.7)	0.28

P-values in bold font indicate statistical significance (P-value <0.05).

**Table 3:** Clinicopathological data among the L1N0, L0N1, and L1N1 group in patients who underwent RP with PLND.

	L1N0 (n = 53)	L0N1 (n = 55)	L1N1 (n = 50)	p-value
Age (mean)	68.3 ± 5.8	67.4 ± 6.9	68.3 ± 7.3	0.57
iPSA	19.2 ± 13.2	30.7 ± 36.7	26.1 ± 19.0	**0.002**
ECOG score 0 1 2	51 (96.2) 1 (1.9) 1 (1.9)	54 (98.2) 1 (1.8) 0	49 (98.0) 1 (2.0) 0	0.736
Operation type Open Laparoscopic Robotic	20 (37.7) 2 (3.8) 31 (58.5)	31 (56.4) 0 24 (43.6)	26 (52.0) 0 24 (48.0)	0.031
pT stage 2a 2b 2c 3a 3b 4	1 (1.9) 0 2 (3.8) 20 (37.7) 29 (54.7) 1 (1.9)	0 0 1 (1.8) 21 (38.2) 31 (56.4) 2 (3.6)	0 0 3 (6.0) 11 (22.0) 35 (70.0) 1 (2.0)	0.490
Gleason Score 6 7 8 9 10	3 (5.7) 23 (43.4) 9 (17.0) 17 (32.1) 1 (1.9)	0 34 (61.8) 10 (18.2) 11 (20.0) 0	0 25 (50.0) 5 (10.0) 20 (40.0) 0	0.068
Positive surgical margin	37 (69.8)	37 (67.3)	39 (78.0)	0.451
Venous invasion	6 (11.3)	4 (7.3)	9 (18.0)	0.236
Perineural invasion	47 (88.7)	51 (92.7)	44 (88.0)	0.681
Adjuvant radiation therapy	0	0	1 (2.0)	0.337
Adjuvant ADT	3 (5.7)	15 (27.3)	10 (20.0)	**0.012**

P-values in bold font indicate statistical significance (P-value <0.05).

### 3.2 Biochemical recurrence-free survival

**Figure 1:**
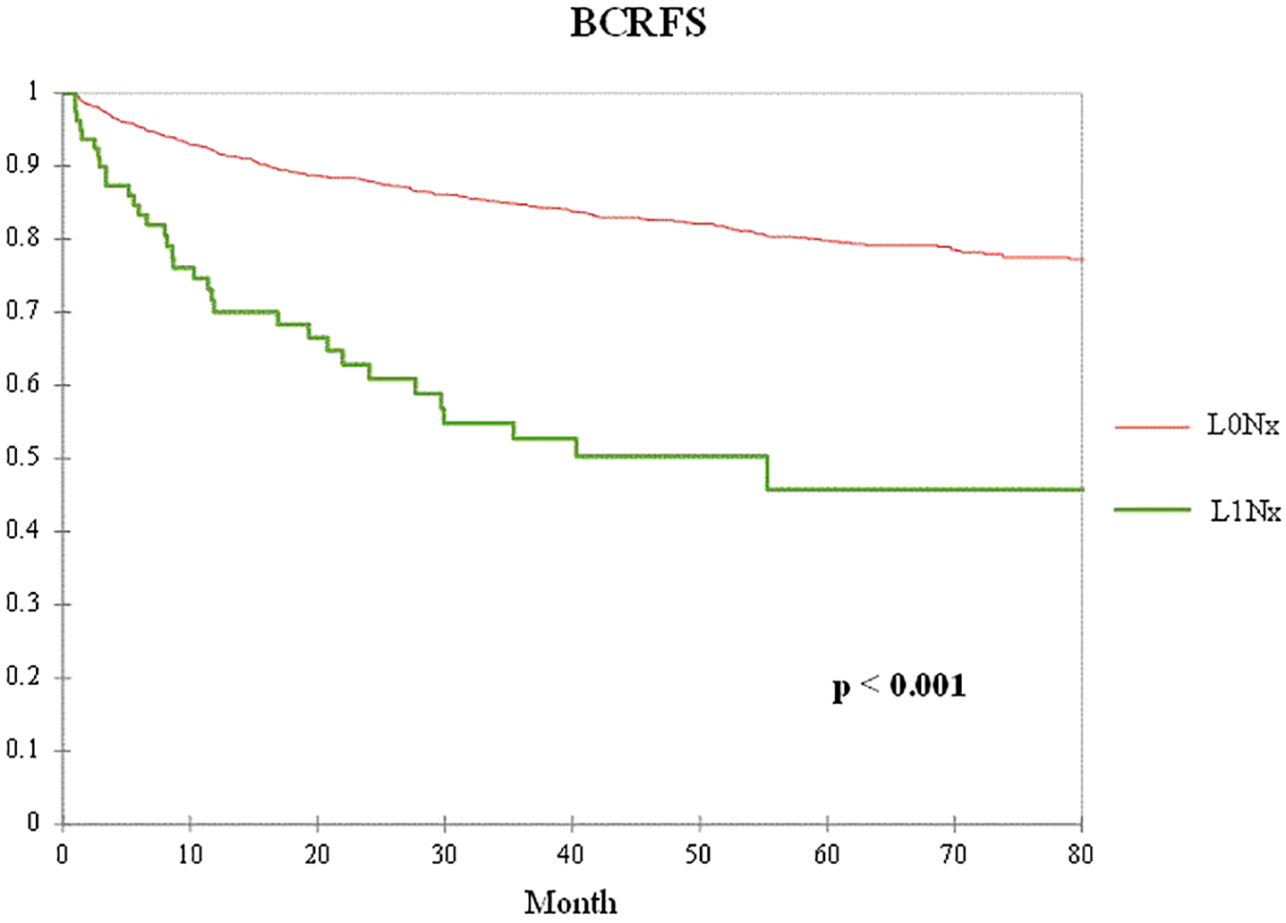
Kaplan–Meier curve of the biochemical recurrence-free survival (BCRFS) between the L0Nx group (red line) and the L1Nx group (green line) in patients who underwent radical prostatectomy (RP) without pelvic lymph node dissection (PLND).

**Figure 1** shows a comparison of biochemical recurrence-free survival (BCRFS) according to the presence or absence of lymphatic invasion after RP without PLND. The L1Nx group showed significantly worse BCRFS than the L0Nx group (p < 0.001).

**Figure 2:**
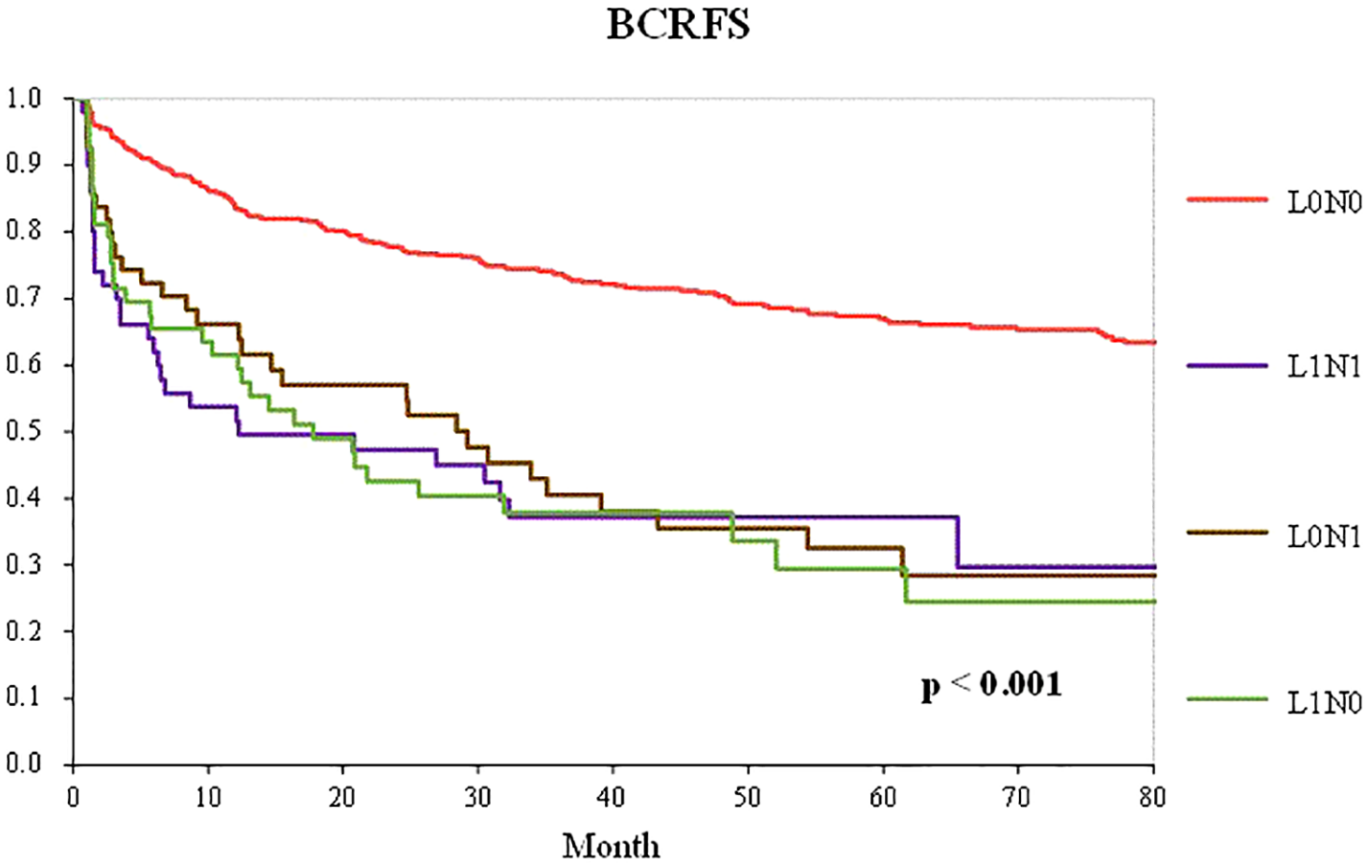
Kaplan–Meier curve of the BCRFS according to the combination of pathological lymph node metastasis and lymphatic invasion status in patients who underwent RP with PLND (L0N0 group—red line; LINO group—green line; LONI group—brown line; LIN1 group—purple line).

**Figure 2** shows BCFRS according to the combination of pathological lymph node metastasis and lymphatic invasion status in patients who underwent RP with PLND. The L1N0, L0N1, and L1N1 groups had significantly worse prognoses than the L0N0 group (p < 0.001). However, there was no significant difference in BCRFS between the L1N0 and lymph node metastasis groups, including the L0N1 and L1N1 groups.

According to the multivariate Cox regression analysis of BCRFS between the L0N0 group and the L1N0 group, lymphatic invasion was identified as a significant predictor of BCR (hazard ratio [HR], 1.64; 95% confidence interval [CI], 1.21–2.02). Moreover, other covariates, including a high Gleason score, pathological T stage, and positive surgical margin also showed a detrimental prognostic impact on BCR (**Table 4**).

**Table 4:** Multivariable Cox regression analysis predicting BCR between L0N0 group and L1N0 group in patients who underwent RP with PLND.

	HR (95% CI)	p-value
Age	0.992 (0.973–1.012)	0.437
iPSA	1.003 (0.999–1.007)	0.198
Gleason Score 6 7 8 9 10	Reference 1.941 (1.008–3.739) 4.149 (2.012–8.557) 3.359 (1.630–6.922) 7.775 (0.954–63.342)	Reference **0.047** **< 0.001** **0.001** 0.055
pT stage 2a 2b 2c 3a 3b 4	Reference 1.988 (0.242–16.319) 2.114 (0.951–4.698) 2.977 (1.333–6.649) 4.517 (1.967–10.369) 5.787 (1.133–29.555)	Reference 0.522 0.066 **0.008** **< 0.001** **0.035**
Positive surgical margin	1.637 (1.209–2.022)	**0.001**
Lymphatic invasion	1.639 (1.110–2.421)	**0.013**
Venous invasion	1.351 (0.584–3.125)	0.482
Perineural invasion	1.153 (0.840–1.583)	0.379

P-values in bold font indicate statistical significance (P-value <0.05).

### 3.3 Comparison after propensity score matching

**Figure 3:**
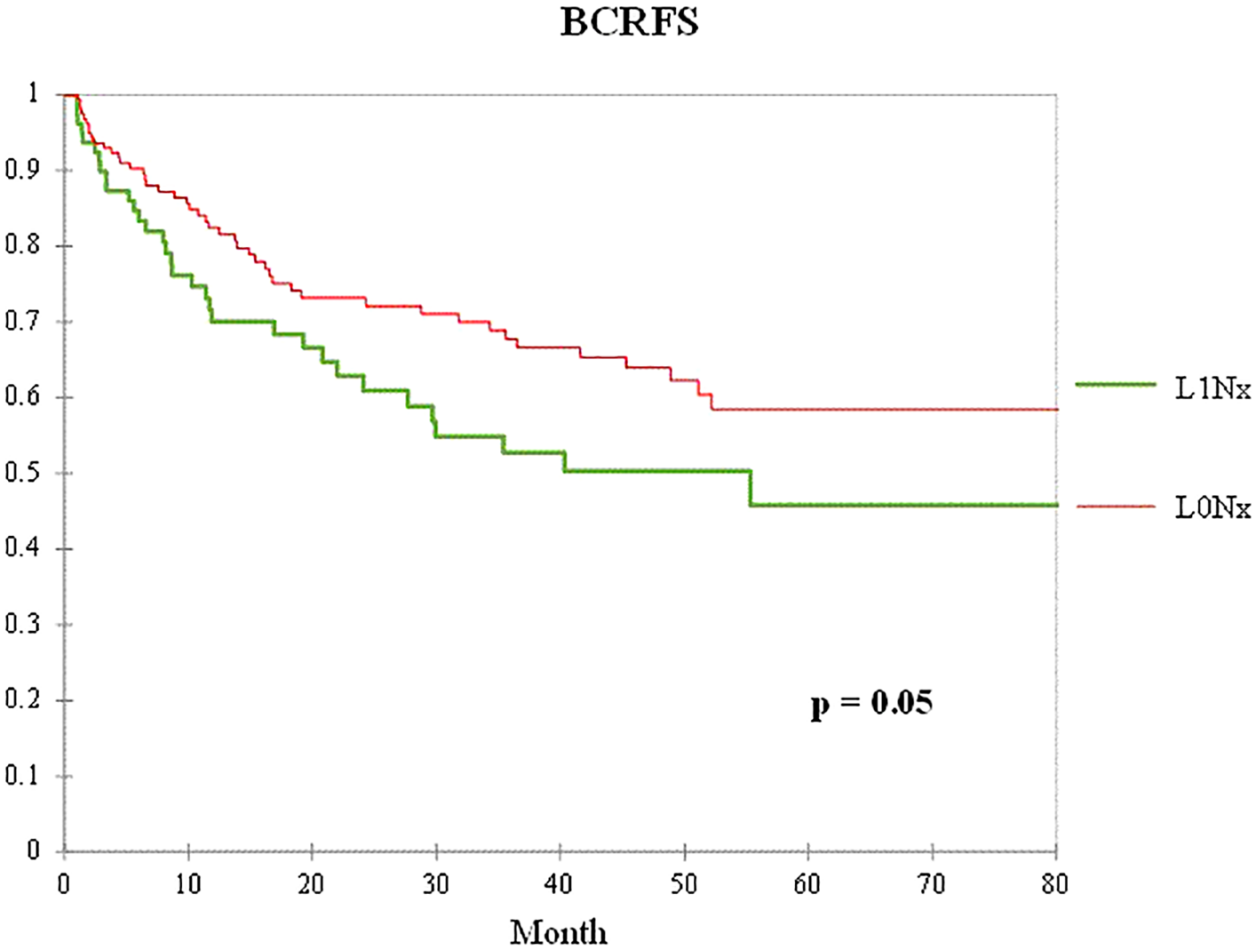
Kaplan–Meier curve of the BCRFS between the L0Nx group (red line) and the L1Nx group (green line) after 1:2 propensity score matching in patients who underwent RP without PLND.

To control for the influence of age, initial serum PSA level, pathological T stage, and Gleason score in patients who underwent RP without PLND, 79 patients in the L1Nx group were matched in a 1:2 ratio with 2,131 patients in the L0Nx group. After propensity score matching, the clinicopathological characteristics of the two groups were well-balanced, with no significant differences among all variables, except perineural invasion (**Table 5**). **Figure 3** shows the Kaplan–Meier curve of the BCFRS between the L0Nx and L1Nx groups after propensity score matching at a 1:2 ratio. Even after propensity score matching, the L1Nx group showed significantly poorer BCRFS than the L0Nx group (p = 0.05).

**Table 5:** Comparison of baseline variables for the between the L0Nx group and the L1Nx group after 1:2 propensity score matching in patients who underwent RP without PLND.

	L0Nx (n = 158)	L1Nx (n = 79)	p-value
Age (mean)	67.2 ± 6.4	66.9 ± 8.2	0.814
iPSA	15.19 ± 17.6	13.89 ± 13.08	0.561
ECOG score 0 1	157 (99.4) 1 (0.6)	79 (100) 0	0.367
Operation type Open Laparoscopic Robotic	65 (41.1) 3 (1.9) 90 (57.0)	36 (45.6) 0 43 (54.4)	0.255
pT stage 2a 2b 2c 3a 3b 4	10 (6.3) 2 (1.3) 31 (19.6) 65 (41.1) 49 (31.0) 1 (0.6)	4 (5.1) 1 (1.3) 17 (21.5) 32 (40.5) 25 (31.6) 0	0.957
Gleason Score 6 7 8 9 10	0 110 (69.6) 9 (5.7) 37 (23.4) 2 (1.3)	0 54 (68.4) 5 (6.3) 18 (22.8) 2 (2.59)	0.913
Positive surgical margin	68 (43.0)	43 (54.4)	0.098
Venous invasion	3 (1.9)	5 (6.3)	0.086
Perineural invasion	97 (61.4)	61 (78.5)	**0.007**

P-values in bold font indicate statistical significance (P-value <0.05).

**Figure 4:**
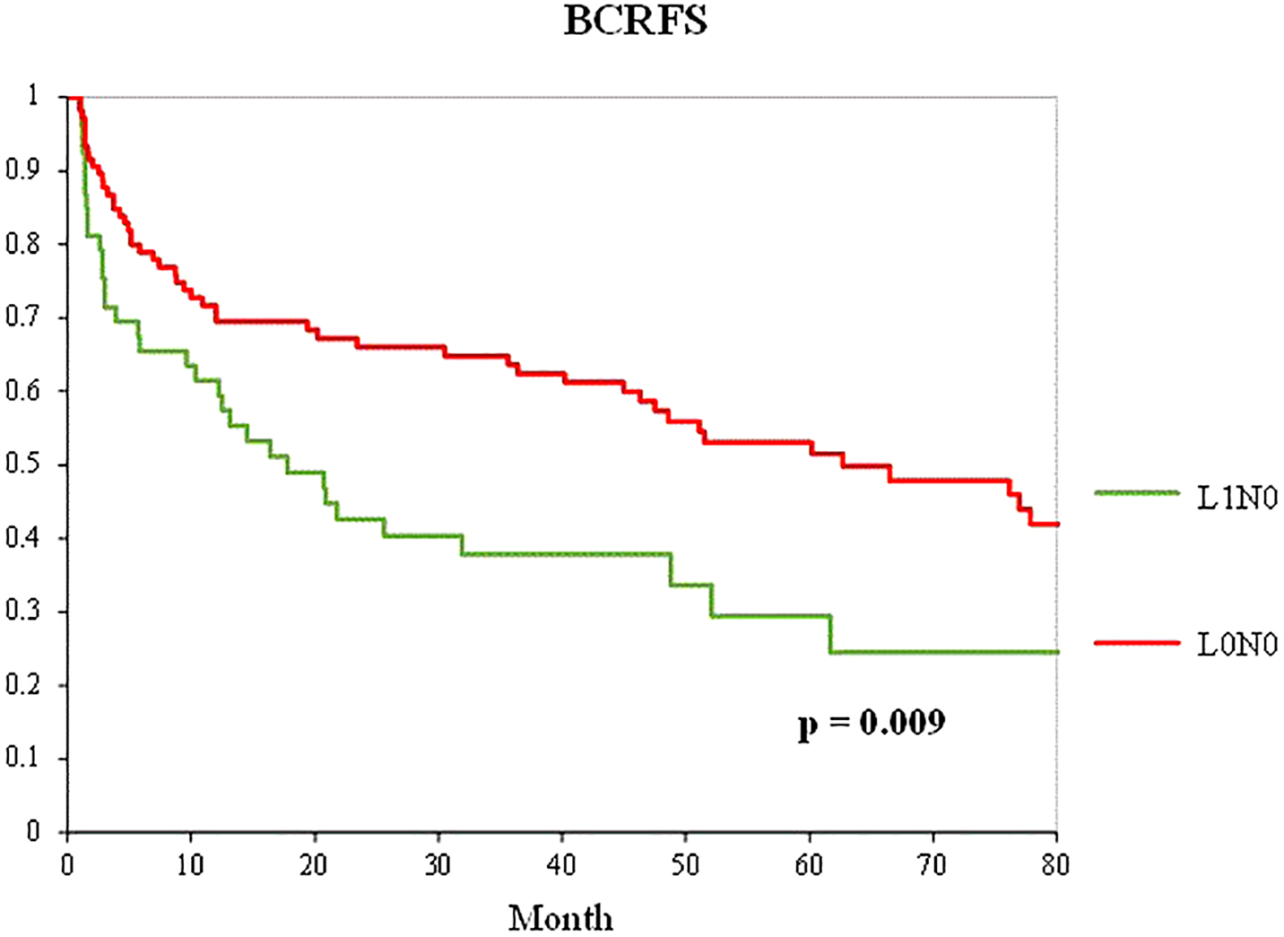
Kaplan–Meier curve of the BCRFS between the L0N0 group (red line) and the L1N0 (green line) group after 1:2 propensity score matching in patients who underwent RP with PLND.

Among the patients who underwent radical prostatectomy with pelvic lymph node dissection, propensity score matching was performed in a 1:2 ratio between 53 patients in the L1N0 group and 584 patients in the L0N0 group. The baseline variables were also well-balanced, with no significant differences among the other variables except for venous invasion (**Table 6**). **Figure 4** shows the Kaplan–Meier curve of the BCFRS after propensity score matching between the L0N0 and L1N0 groups. The L1N0 group showed a significantly worse prognosis than the L0N0 group after propensity score matching (p = 0.009).

**Table 6:** Comparison of baseline variables for between the L0N0 group and the L1N0 group after 1:2 propensity score matching in patients who underwent RP with PLND.

	L0N0 (n = 106)	L1N0 (n = 53)	p-value
Age (mean)	69.1 ± 6.02	68.3 ± 5.76	0.434
iPSA	20.9 ± 22.3	19.2 ± 13.2	0.612
ECOG score 0 1 2	106 (100) 0 0	51 (96.2) 1 (1.9) 1 (1.9)	0.108
Operation type Open Laparoscopic Robotic	53 (50.0) 2 (1.9) 51 (48.1)	20 (37.7) 2 (3.8) 31 (58.5)	0.303
pT stage 2a 2b 2c 3a 3b 4	3 (2.8) 0 3 (2.8) 44 (41.5) 54 (50.9) 2 (1.9)	1 (1.9) 0 2 (3.8) 20 (37.7) 29 (54.7) 1 (1.9)	0.978
Gleason Score 6 7 8 9 10	6 (5.7) 44 (41.5) 22 (20.8) 33 (31.1) 1 (0.9)	3 (5.7) 23 (43.4) 9 (17.0) 17 (32.1) 1 (1.9)	0.97
Positive surgical margin	63 (59.4)	37 (69.8)	0.198
Venous invasion	0	6 (11.3)	**<0.001**
Perineural invasion	83 (78.3)	47 (88.7)	0.099

P-values in bold font indicate statistical significance (P-value <0.05).

## 4 Discussion

In the present study, we analyzed the risk of BCR in the presence of lymphatic invasion among patients who underwent RP without PLND. We identified significant differences in BCRFS between the L0Nx and L1Nx groups before and after propensity score matching. Second, we evaluated the effects of lymphatic invasion on BCR with respect to lymph node metastasis in patients who underwent RP with PLND. The lymphatic invasion without lymph node metastasis (L1N0) group showed similar BCRFS to the pN1 group in the Kaplan–Meier curve. Furthermore, after propensity score matching between the L0N0 group and the L1N0 group, lymphatic invasion was the only significant predictor of BCR. BCRFS also showed a significant difference between the two groups after propensity score matching.

Most previous studies have demonstrated the prognostic impact of lymphovascular invasion in prostate cancer, which integrates both lymphatic and venous invasion. In the present study, we separated lymphatic and venous invasions from lymphovascular invasion. A few recent studies have analyzed the prognostic impact of lymphatic invasion alone. Wilczak et al. retrospectively examined 14,528 patients who underwent radical prostatectomy and studied the prognostic value of lymphatic invasion (16). It was concluded that lymphatic invasion provides prognostic information comparable to that of lymph node status. However, there was a huge gap in the number of patients between the two groups, and no balance of covariates was achieved between them. Thus, we performed propensity score matching to overcome the limitations of this retrospective observational study. Yamashita et al. prospectively analyzed 183 consecutive patients with high-risk prostate cancer who underwent robot-assisted radical prostatectomy with extended lymph node dissection and showed that lymphatic invasion was an independent significant predictor of BCR (17). Despite the advantages of this prospective study, the sample size was small, and the follow-up period after surgery was relatively short.

Although propensity score matching performed well, we obtained one question about our results. Perineural (**Table 5**), venous (**Table 6**), and lymphatic invasion also showed significant differences. Several studies have demonstrated that perineural invasion in prostate cancer is an independent prognostic factor (18, 19); however, to the best of our knowledge, no studies have investigated the prognostic significance of venous invasion alone. There may be a correlation between perineural, venous, and lymphatic invasion; hence, further studies are required to validate this association.

This study has some limitations. First, we retrospectively analyzed the patients. Second, several surgeons performed the radical prostatectomies. Third, clinical data were obtained from a single center. Fourth, we did not incorporate pathological variants such as the cribriform pattern and intraductal carcinoma of the prostate in our study. Fifth, the number of patients received adjuvant treatment is too few to be used as accurate factor. To overcome these limitations, a randomized prospective trial including pathological variants is required to verify the results of the present study.

## 5 Conclusion

Lymphatic invasion in radical prostatectomy specimens is an independent prognostic factor, which can complement lymph node status for predicting biochemical recurrence. Considering lymphatic invasion as an adverse pathologic finding, similar to lymph node metastasis, adjuvant therapy could be considered in patients with lymphatic invasion.

## Data availability statement

The original contributions presented in the study are included in the article/supplementary material. Further inquiries can be directed to the corresponding author.

## Ethics statement

The studies involving humans were approved by Institutional Review Board (IRB No. H-2305-041-1430) of Seoul National University Hospital. The studies were conducted in accordance with the local legislation and institutional requirements. The ethics committee/institutional review board waived the requirement of written informed consent for participation from the participants or the participants’ legal guardians/next of kin because retrospective nature of the study.

## Author contributions

Conceptualization: DC. Data collection: DC, S-HJ, JH, and HY. Data analysis DC, S-HJ, and CK. Data visualization: DC and S-HJ. Data interpretation: DH, S-HJ, JH, HY, CJ, JK, and CK. Manuscript writing: DC. Supervision: DC, S-HJ, and CK. All authors contributed to the article and approved the submitted version.
